# The osteocalcin gene rs1800247 polymorphism in Kashubian population

**DOI:** 10.1007/s00296-012-2575-1

**Published:** 2012-12-12

**Authors:** Krzysztof Specjalski, Maria Porzezińska, Alicja Siemińska, Jan M. Słomiński, Ewa Jassem

**Affiliations:** 1 Department of Allergology, Medical University of Gdansk, ul. Debinki 7, 80-952 Gdansk, Poland; 2 Department of Pneumonology, Medical University of Gdansk, Gdansk, Poland

**Keywords:** Osteocalcin, Single nucleotide polymorphism, Osteoporosis, Kashubia

## Abstract

Osteocalcin is the most important noncollagenous protein component of the bone. Polymorphisms of osteocalcin gene were reported to be associated with bone mineral density. However, this relation was only confirmed in some populations. In this study presence of C/T polymorphism in osteocalcin gene (rs1800247) was determined in Kashubian population (northern Poland). The frequencies of variants were CC 9 %, TC 31 %, and TT 60 %, with no significant differences between genders. The genotypes were in Hardy–Weinberg equilibrium.

## Introduction

Osteoporosis is a multifactorial skeletal disease leading to loss of bone mass and deterioration of its micro-architecture with consequent pathologic fractures. In the United States, Europe, and Japan, it touches 75 million people. It is estimated that worldwide up to 50 % of women and 30 % of men will experience low-energy fracture during their lifetime [1]. In Poland osteoporosis is found in 18.5 % and osteopenia in 40 % of postmenopausal women [2]. It has been shown that bone mineral density (BMD) and susceptibility to fractures are partly influenced by polymorphisms of numerous genes. The candidates included genes coding: vitamin D receptor, estrogen receptor, collagen type 1, interleukin 6, and osteocalcin [3, 4].

Osteocalcin is the most important noncollagenous protein component of the bone, secreted by osteoblasts. It is considered to be a good marker of bone turnover and osteoblast activity. Dohi et al. [5] have shown that HindIII polymorphism of osteocalcin gene was associated with osteopenia in postmenopausal Japanese women. This relation was also confirmed in Swedish population [6]. Recently, polymorphism rs1800247 was found to be associated with the serum osteocalcin concentration and rs1543297—with the risk of fracture [7]. On the other hand, in Chinese population no association has been found between the polymorphism and bone mineral density [8, 9].

For numerous single nucleotide polymorphisms, it has been shown that frequency of allelic variants in several parts of the world is diverse [10]. The region of Kashubia, located in northern Poland, is inhabited by the relatively homogeneous ethnic group genetically different from the general Polish population [11]. Polymorphisms in osteocalcin promoter have never been determined in this region.

The object of the present study was to determine the prevalence of osteocalcin promoter gene rs1800247 polymorphism in Kashubian population. This is the first stage of a study aiming at investigating relations between genetic polymorphisms and development of steroid-induced unwanted effects.

## Patients and methods

A total of 250 subjects, including 110 women and 140 men, were randomly chosen from the population of Kashubia region (northern Poland). Study participants were aged 18–85 (mean age—46,2 years).

For the genetic analysis peripheral blood was collected (5 ml) and stored at the temperature of −70 °C. DNA was isolated from 100 μl frozen blood with the use of *Genomic Micro AX Blood Gravity* (*A&A Biotechnology*, Gdynia, Poland) according to manufacturer’s instructions.

Fragments of genomic DNA containing SNPs were amplified with forward and reverse primers: 5′-CCGCAGCTCCCAACCACAATAAGCT-3′, 5′-CAATAGGGCGAGGAGT-3′, respectively. The reaction was performed in the mixture of 20 μl containing 2×PCR Master Mix Plus High GC (*A&A Biotechnology,* Gdynia, Poland)—10 μl, genomic DNA—0.5 μl, forward primer (100 μM)—0.1 μl, reverse primer (100 μM)—0.1 μl, and DEPC—9.3 μl. Temperature cycles in PCR were as follows: 94 °C—3 min., 30× (denaturation at 94 °C—30 s, annealing at 56 °C—30 s, elongation at 72 °C—30 s), 72 °C—5 min.

Products of amplification were digested with restrictive enzymes. The reaction was conducted at 37 °C, in the 20 μl mixture containing PCR product—5 μl, reaction buffer—2 μl, restrictive enzyme (*Fermentas*, St. Leon-Rot, Germany)—0.5–1 μl, DEPC—20 μl.

Products of digestion were separated in 2 % agarose gel, in TAE buffer with the voltage of 7 V/cm.

In statistical analysis χ^2^ test was used to check whether the variants were in Hardy–Weinberg equilibrium.

## Results

Presence of C/T polymorphism was found in osteocalcin gene (rs1800247). The frequencies of variants were as follows: CC 9 %, TC 31 %, and TT 60 %, with no significant differences between genders. The genotypes conformed to Hardy–Weinberg equilibrium.

## Discussion

The current findings suggest that tendency to develop osteoporosis may have a genetic background, with a possible role of polymorphisms of numerous genes [3, 4]. Some of them, including osteocalcin gene, were reported to be associated with bone mineral density. However, this relation was only confirmed in some populations [5–9].

Demonstrating the role of genes in the pathogenesis of osteoporosis requires investigating the presence of polymorphic variants in the population tested and finding their correlation with the clinical outcome. This study addresses the first issue showing the distribution of polymorphic variants in the population of Poland. The frequencies of allelic variants are similar to other Caucasian and Chinese populations [6, 8]. However, their impact on bone mineral density needs to be confirmed.

In conclusion, further studies need to be conducted to confirm the association between BMD, risk of fractures, and rs1800247 polymorphism.
